# Oligometastatic NSCLC: Current Perspectives and Future Challenges

**DOI:** 10.3390/curroncol32020075

**Published:** 2025-01-29

**Authors:** Sara Torresan, Jacopo Costa, Carol Zanchetta, Lorenzo De Marchi, Simona Rizzato, Francesco Cortiula

**Affiliations:** 1 Department of Medicine (DME), University of Udine, 33100 Udine, Italy; sara.torresan@cro.it (S.T.);; 2 Department of Medical Oncology, IRCCS, Centro di Riferimento Oncologico CRO di Aviano, 33081 Aviano, Italy; 3 Department of Oncology, University Hospital of Udine, 33100 Udine, Italy; 4 Department of Respiratory Medicine, Maastricht University Medical Centre, GROW School for Oncology and Reproduction, 6229 ER Maastricht, The Netherlands

**Keywords:** NSCLC, oligometastatic, oligoprogressive, oncogene-addicted NSCLC, non-oncogene-addicted NSCLC

## Abstract

Oligometastatic non-small cell lung cancer (NSCLC) represents a separate entity with a different biology and prognosis compared to stage IV NSCLC. Challenges range from the very definition of oligometastatic disease to the timing and techniques of local treatments, and their benefit in prolonging patient survival. Most of the international consensus and guidelines agree on the need for shared criteria, such as appropriate stadiation and even tissue biopsy if needed, in order to select patients that could really benefit from personalised strategies. Multidisciplinary evaluation is crucial in order to define if every lesion is amenable to radical local treatment, which appears to be the most important criterion across different guidelines. A distinction must be made depending on the time of oligo-disease detection, separating de novo oligometastatic disease from oligorecurrence, oligoprogression and oligoresidual disease. These separate entities imply a different biology and prognosis, and treatment strategies consequently must be tailored. Locoregional approaches are therefore often contemplated in order to ensure the best outcome for the patient. In non-oncogene-addicted disease, the advent of immune checkpoint blockers (ICBs) allows physicians to take into consideration consolidative treatments, but timing, technique and subsequent systemic treatment remain open issues. In oncogene-addicted NSCLC, local treatments are nowadays preferably reserved to cases of oligoprogression, but the advent of new, more potent drugs might challenge that. In this review, we summarised the current knowledge, consensuses and data from retrospective and prospective trials, with the aim of shedding some light on the topic and emphasising the unmet clinical need.

## 1. Introduction

The concept of oligometastatic disease was first introduced by Hellman et al. in 1995 [1], who defined it as an intermediate status between localised disease and the presence of multiple metastatic sites. In non-small cell lung cancer (NSCLC), oligometastatic disease represents approximately one third of all metastatic disease diagnoses [2]. Patients with oligometastatic disease usually have a better median overall survival (mOS) compared to diffuse NSCLC [3,4]. The TNM AJCC 8th edition does recognize the different prognosis of oligometastatic disease, classifying M1b disease as that limited to one single extrathoracic metastasis (stage IVA) [5]. The most common sites of oligometastases are usually the brain, lung and adrenal gland, followed by the bones [6]. However, a shared and standardised definition of oligometastatic disease has only been achieved recently. Thus, studies involving oligometastatic NSCLC (omNSCLC) are not easily comparable since the inclusion criteria differ.

**Figure 1:**
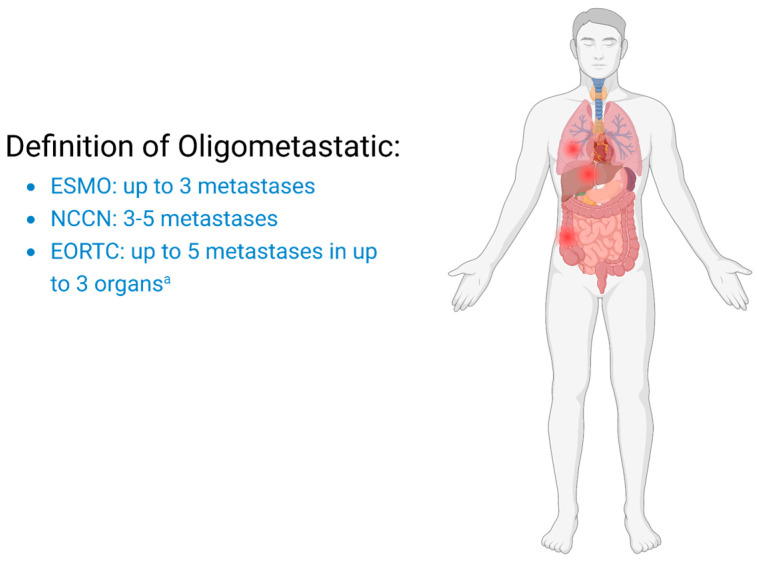
Oligometastatic disease defined by the ESMO, NCCN and EORTC. A commonly accepted and recognised definition. Abbreviations: ESMO = European Society of Medical Oncology; NCCN = National Comprehensive Cancer Network; EORTC = European Organization for Research and Treatment of Cancer.

According to the European Society of Medical Oncology (ESMO), the most accepted definition of oligometastatic disease comprehends at most five metastatic sites [7], whereas the National Comprehensive Cancer Network (NCCN) proposes three to five metastatic sites [8] (Figure 1).

The European Organization for Research and Treatment of Cancer (EORTC) promoted a survey demonstrating how physicians’ perceptions of omNSCLC differed significantly with regard to the number of metastases, the number of organs involved, or the consideration of mediastinal lymph nodes [2]. Following this survey, a consensus definition was reached stating that omNSCLC could be defined by the presence of up to five metastases in up to three organs [7]. Notably, 15% of physicians stated that the number of organs involved was not significant as long as local radical treatment was feasible. No consensus was reached regarding the volume of metastases, histology differences or genomic background [9].

After reviewing the literature and considering the survey results, the International Association for the Study of Lung Cancer (IASLC) also proposed a similar definition of synchronous oligometastatic disease (up to five metastases in up to three organs) [7]. On top of that, the IASLC stated that to be defined as oligometastatic the disease has to be radically treatable. In both definitions, mediastinal lymph nodes were not considered metastatic sites, nor was the size of the metastasis taken into account [7,9]. Another question is whether to classify bilateral synchronous lung nodules as two primary tumours or as metastatic disease [10].

Based on the timing of oligometastatic disease, some authors have proposed different definitions: synchronous, metachronous, oligoresidual and oligoprogressive disease. The first one refers to de novo oligometastatic disease. Metachronous omNSCLC, on the other hand, applies to previously radically treated localised NSCLC which recur in few, potentially radically treatable sites [11]. Oligoresidual disease refers to the few metastatic sites that remain (radiologically) after an effective systemic treatment, while oligoprogression describes a disease that under systemic treatment only progress in a few sites [11].

The aim of this review is to summarise the current evidence, the challenges and the future perspectives for patients with omNSCLC.

## 2. Discussion

In clinical practice, omNSCLC is considered to be halfway between localised and plurimetastatic disease. Locoregional approaches are therefore often contemplated in order to optimise systemic treatment duration and sequence [12]. In fact, systemic treatment frequently remains the backbone of the treatment plan [13,14]. Careful timing of different treatment approaches, as well as the best technique for locoregional treatments, are key requirements to ensure the best outcome for the patient.

Most data about the treatment of omNSCLC are derived from retrospective studies, since clinical trials specifically for patients with omNSCLC have only been developed in recent years. Moreover, the variable definition of omNSCLC among different studies and different inclusion criteria (e.g., metachronous and synchronous) prevents researchers from drawing firm conclusions.

ESMO clinical guidelines recommend systemic therapy plus local ablative treatment (LAT) irrespective of molecular profiling. However, they do not give a definitive indication about the timing of LAT [13]. NCCN recommends definitive/consolidative treatment to both metastatic sites and the primary site [8]. More importantly, they recommend local consolidative treatment (LCT) in patients who do not progress on systemic treatment, hinting at the best possible timing of the different approaches. In particular, these guidelines underline the role of stereotactic ablative radiotherapy (SABR) as the definitive treatment technique. Recently, the American Society for Radiation Oncology (ASTRO) published the first guidelines specifically dedicated to omNSCLC [12]. They recommend LCT when all disease sites are amenable to radical treatment and exclude all local treatment modality except surgery and radiotherapy (like ablative techniques). They also recommend maintenance treatment after LCT, particularly for synchronous omNSCLC. However, upfront local treatment is still suggested for symptomatic lesions and for patients not suitable for or who refuse systemic therapy [15]. Furthermore, local re-treatment might be considered in patients with oligoprogression after previous LCT [12].

Importantly, an accurate staging with positron emission tomography (PET-FDG) and brain magnetic resonance imaging (MRI) is recommended. Furthermore, if only a single metastatic site is identified, histologic confirmation is recommended, even in cases of mediastinal involvement if it impacts treatment choice [13].

A phase II study (N = 29) showed a progression-free survival (PFS) improvement (9.7 vs 3.5 months, *p* = 0.01) with the addition of SABR to all sites of disease on top of maintenance chemotherapy, compared to maintenance chemotherapy alone, after induction chemotherapy [16]. Patients with up to five metastases were eligible, and partial response or stable disease after 4–6 cycles of induction chemotherapy was required. The study was stopped prematurely after an interim analysis. None of the patients enrolled received ICB. In a phase II randomised clinical trial (RCT) (N = 49), patients with up to three metastases were randomly assigned to either LCT (with optional subsequent maintenance) or to maintenance or observation alone, after at least 3 months of chemotherapy [17]. Patients treated with LCT presented a better PFS (11.9 [90% confidence interval (CI) 5.7–20.9] vs 3.9 months (2.3–6.6), hazard ratio (HR) 0.35, *p* = 0.0054) and better OS (mOS 41.2 [95% CI, 18.9—not reached (NR)] vs 17.0 months [95% CI, 10.1 to 39.8 months]; *p* = 0.017) [18]. Again, none of the patients received ICB.

A prospective single-arm phase II trial (N = 40) on synchronous omNSCLC evaluated LCT (surgery or radiotherapy to all sites) on top of systemic therapy (chemotherapy 95%, none ICB). No previous response to systemic treatment was required. Median OS was 13.5 months (95% CI 7.6–19.4) and median PFS (mPFS) was 12.1 months (95% CI 9.6–14.3). Only 12.9% of patients had more than one metastasis [19,20]. Another prospective, single-arm phase II trial by Arrieta et al. (N = 37) evaluated SABR, administered after four cycles of induction systemic therapy, in patients with non-progressive synchronous omNSCLC. After SABR, all patients received maintenance therapy. In this study, 43.2% of 37 patients had central nervous system (CNS) metastases. Patients with up to five metastases were eligible. Notably, following SABR, 51.5% of the patients had complete radiometabolic response, with a not reached mOS [21].

The phase II RCT SABR-COMET (N = 99, N = 18 with NSCLC) included patients with up to five metachronous metastases. Patients were randomised to receive SABR to all metastatic lesions on top of systemic treatment (the trial was conducted before the approval of ICB). Both mPFS and mOS were significantly longer in the SABR arm: 11.6 vs 5.4 months (*p* = 0.001; HR 0.48; 95% CI 0.31–0.76) and 50 vs 28 months (*p* = 0.006; HR 0.47; 95% CI 0.27–0.81), respectively [22].

In a phase II study by Petty et al. (N = 29), patients with metachronous omNSCLC were treated with consolidation radiotherapy to all sites after achieving at least stable disease with 3–6 cycles of chemotherapy [23]. The study was prematurely stopped because of slow accrual but met its primary endpoint of PFS >6 months (mPFS 11.2 months, 95% CI 7.6–15.9 months, *p* < 0.0001). The mOS was 28.4 months (95% CI 14.5–45.8). In this study, radiotherapy was followed by observation alone.

In another prospective, single-arm phase II trial, systemic treatment (target therapy or chemotherapy) followed by consolidative SABR to all sites in non-progressive omNSCLC was investigated [24]. Disease control rate (DCR) and complete metabolic response (assessed by 18F-FDG-PET/CT) to SABR were 93.6% and 70.2%, respectively. Patients with a complete metabolic response had a median PFS of 53.9 months vs 31.9 months in those who did not achieve it (*p* = 0.011). In this study only patients with intrapulmonary metastases were included.

### 2.1. Oligometastatic NSCLC in the Immunotherapy Era

In the era of ICB, useful information could be retrieved from registrative phase III RCTs about the efficacy of systemic treatment alone (in terms of PFS and OS). This particularly pertains to understanding the need for LCT in addition to ICB. However, neither the number of metastatic sites of the patients nor the number of omNSCLC was described in any of the studies [25,26,27,28].

A prospective analysis of patients with borderline resectable or omNSCLC (N = 22/86) investigated the efficacy of resection/definitive chemoradiotherapy (CRT) after (chemo)-immunotherapy. The primary endpoint was the proportion of patients receiving curative treatment, reached by 21 omNSCLC patients. Median PFS was not reached in patients who underwent surgery, while it was 11.9 months in patients who received CRT [29].

In an observational retrospective, single-centre analysis, the addition of LCT to pembrolizumab (plus chemotherapy when indicated) in 251 patients with omNSCLC showed an mPFS in the LCT and non-LCT arms of 13.97 and 10.08 months, respectively (HR 0.64 *p* = 0.016), and an mOS of 30.67 and 21.97 months (HR 0.53 *p* = 0.011), respectively [30].

In a single-arm phase II trial, local ablative treatment was administered upfront to patients with both synchronous and metachronous omNSCLC. Afterwards, if no disease progression occurred, patients received pembrolizumab. Median PFS from the start of local treatment was 19.1 months and mOS was 41.6 months. Notably, 28 of the 45 patients had only one metastasis [31]. In a phase II study (N = 35), patients with omNSCLC received chemo-immunotherapy followed by SABR or radiotherapy (RT), with subsequent maintenance with durvalumab [32]. Median PFS was 10.4 m (95% CI 4.4-NR) with an objective response rate (ORR) of 71.9%.

Recently, the results of the phase II/III NRG-LU002 1 study were presented [33]. Patients (N = 215) with omNSCLC were randomised to LCT plus maintenance and systemic treatment vs systemic treatment alone, after four cycles of induction chemo-immunotherapy, with no evidence of progressive disease (PD) (regardless of PDL-1). The final results were not clinically meaningful: one-year and two-year PFSs (primary endpoint) for standard and experimental arms were 48% vs 52% and 36% vs 40%, respectively (*p* = 0.66, HR 0.93 (95%CI 0.66–1.31)). The OS analysis reported similar results with a HR of 1.05 (95% CI 0.70–1.56).

An important limitation of all these trials is that not all trials mandated baseline PET and brain imaging, which are recommended in patients with oligometastatic disease and could aid in better selecting patients and thus improving outcomes.

A potential advantage of stereotactic body radiation therapy (SBRT) coupled with ICB is the possible abscopal effect. Welsh et al. [34], in a phase I/II MDACC trial, explored the safety and efficacy of pembrolizumab with or without concurrent SBRT to liver and lung metastases, in patients with at least one lesion in another organ. The primary endpoint was the out-of-field objective response rate (ORR) (38% in the pembrolizumab plusSBRT group and 10% in the pembrolizumab + traditional RT group) [35]. Beyond the abscopal effect, it is believed that radiotherapy in general could enhance tumour response to ICB by activating pro-inflammatory pathways, as demonstrated in the randomised phase II study PEMBRO-RT that enrolled 92 patients with advanced NSCLC and evaluated pembrolizumab after radiotherapy to a single tumour site or alone. Better ORRs (primary endpoint) and PFSs were achieved in the experimental arm in the PDL-1 negative subgroup: 12 weeks ORR 36% vs 18%, mPFS 1.9 months (1.7–6.9 months) vs 6.6 months (4.0–14.6) (HR 0.71; 95% CI 0.42–1.18; *p* = 0.19), mOS 7.6 months (6.0–13.9) vs 15.9 months (7.1-NR) (HR 0.66; 95% CI 0.37–1.18; *p* = 0.16), but these were not statistically significant [36].

An analysis combining these two trials (MDACC and PEMBRO-RT) showed an mOS of 8.7 months (6.4–11.0) with pembrolizumab vs 19.2 months (14.6–23.8) with pembrolizumab plus RT (HR 0.67, 0.54–0.84; *p* = 0.0004) [37]. Interestingly, the abscopal effect was observed in these patients after RT, with an out-of-field response rate of 41.7% [37].

In conclusion, to address the appropriate treatment for this subpopulation of patients, properly designed phase III trials are needed in order to determine the real benefit of LCT and LAT in patients achieving a good response with ICB and the right timing and techniques of local treatments. The small subset of oligometastatic patients who can benefit from this multi-modal approach still needs to be defined, and LAT can be considered as an optimization of an individual patient’s treatment rather than as a standard clinical practice (Table 1).

### 2.2. Real World Data on omNSCLC and Immunotherapy

Despite the scarcity of prospective, randomised trials, omNSCLC is subject to the interest of academic research due to its clinical relevance. Hence, different real-world experiences have been shared in an attempt to supplement available data. The most relevant are summarised in Table 2.

A German retrospective study evaluating patients with synchronous omNSCLC and treated with concomitant CRT (N = 52) showed a 5-years OS rate similar to patients with stage III NSCLC who received cCRT: 28.3%, (95%-CI: 16.4–41.5%) vs 34.9% (95%-CI: 27.4–42.8%) [38]. This study could be relevant for its stratification of omNSCLC patients into two risk groups based on severe comorbidity, ECOG performance status, gender and pre-treatment serum C-reactive protein level, with significant survival differences. A 4-year survival rate of 49.4% was reached in the good prognosis group, while 4-year survival was 9.9% in the poor prognosis group (*p* = 0.0021) [38]. This could help in selecting patients who could benefit from LAT.

A retrospective German study (N = 13) showed the efficacy of chemo-immunotherapy in patients with omNSCLC before resection of the primary site and LAT to metastatic sites. In this study, the pathological complete response and major pathological response were 54 and 69%, respectively [39]. In a retrospective multicentre real-world analysis, 26 patients with omNSCLC received neoadjuvant chemo-immunotherapy followed by resection or SABR to all sites and subsequent consolidation immunotherapy. On surgical specimens, 12 patients achieved pCR [40]. In another retrospective analysis of 68 patients with synchronous omNSCLC who received ICB, 56% of them received LAT on top of ICB systemic treatment [41]. Compared to a historical cohort receiving chemotherapy, patients receiving ICB had a better PFS (19.0 vs 6.8 months, HR 0.5, *p* = 0.03). At the same time, this study suggested that the benefit of adding LAT on top of systemic ICB is not clear: the median PFS was 19.0 months with or without LAT [41].

A German retrospective cohort study (N = 218) showed that patients who had LAT to all sites had an mOS of 34.4 months, and systemic treatment was associated with a doubling of the relapse-free survival (RFS) (12.3 vs 6.4 months, *p*  <  0.001). More interestingly, the addition of ICB to chemotherapy improved the 2-year RFS from 15% to 51% (HR 0.44, *p*  =  0.008) [42].

Evaluating the addiction of immunotherapy in the context of omNSCLC, combined analyses of American and Chinese cohorts (N = 827) showed a different survival benefit according to the organ involved in the metastatic lesions. A better outcome was seen in patients with brain and multi-organ sites, as opposed to liver and bone metastases (HR for OS respectively 0.565/*p* = 0.004, 0.565/*p* = 0.004, 0.776/*p* = 0.303 and 1.24/*p* = 0.229) [43].

Real-world studies and academic research represent an opportunity, both for patients and for clinicians, to ensure a more personalised but evidence-based treatment. Efforts to come together in order to accumulate unbiased, robust data with larger numbers should be encouraged.

### 2.3. Oncogene-Addicted omNSCLC

NSCLC harbour actionable genomic alterations (AGAs) in about 40% of cases, and survivals achieved with target therapies might be of several years [44,45,46]. A specific approach for oncogene-addicted omNSCLC is worthy of investigation. We know that in the majority of patients, at some point the tumour develops a resistance mechanism which would require a new treatment. Lowering the tumour load through a local treatment may postpone the development of a resistance mechanism and thus the need to change systemic therapy [47].

However, even in oncogene-addicted NSCLC settings, RCTs specifically focusing on oligometastatic disease are lacking, and data regarding oncogene-addicted omNSCLC are very scarce, mainly derived from subgroup analyses and retrospective series.

The only available phase III randomised controlled trial specifically enrolling epidermal growth factor receptor (*EGFR*)-mutated synchronous omNSCLCs is the SINDAS trial, in which patients were randomised to receive first-generation tyrosine kinase inhibitors (TKIs) with or without upfront SABR to all sites [48]. An interim analysis showed an mPFS of 12.5 months vs 20.2 months (*p* < 0.001), and an mOS of 17.4 months vs 25.5 months (*p* < 0.001). However, the primary endpoint of a 6-month PFS was negative (99.1% vs 95.2%), and patients with brain metastases were excluded, contributing to a very high screening failure rate (78%) and introducing an important selection bias. Furthermore, FDG-PET was not mandatory to confirm oligometastatic disease.

A retrospective study including patients with *EGFR*-mutated omNSCLC (with metastases in only one organ) (N = 231) showed an mPFS of 15 with the addition of LCT vs 10 months with TKI only (HR 0.61 *p* = 0.000) and an mOS of 34 vs 21 months (HR 0.59, *p* = 0.001) [49]. Another retrospective study (N = 145) enrolled patients with synchronous, *EGFR*-mutated omNSCLC who received a first-generation TKI (erlotinib, gefitinib or icotinib) and had disease control [50]. The study investigated LAT to all, some or no sites of disease. Patients who received LAT to all sites had a better PFS and OS compared to the other groups (mPFS 20.6 vs 15.6 vs 13.9 months respectively, *p* < 0.001; mOS 40.9 vs 34.1 vs 30.8 months respectively, *p* < 0.001).

Small, prospective, phase II studies show survival improvement by the addition of LAT after obtaining a response to systemic treatment [24]. In the already cited study by Gomez et al. and by Parikh et al., patients with an *EGFR* mutation or anaplastic lymphoma kinase (*ALK*) rearrangement were allowed [4,18]. In the former, (N = 8/49), AGAs were linked to a lower risk of death (HR, 0.12 *p* = 0.041). In the latter (N = 20), LAT on the primary tumour and *EGFR* mutation were associated with prolonged OS (HR: 0.65; *p* = 0.043 and HR 0.46; *p* = 0.001, respectively).

The ATOM trial (N = 16) included specifically *EGFR*-mutated omNSCLC, and it also included oligoresidual disease [51]. After 3 months of TKI, 18 patients received LAT, lowering the risk of disease progression (HR 0.41, *p* = 0.0097). However, the trial was stopped due to slow accrual.

The role of SBRT was retrospectively investigated in cohorts of patients with liver [52] (N = 43) and brain [53] (N = 351) metastases in *EGFR*-mutated NSCLC, confirming the OS benefit of TKIs and LAT compared to TKI alone (mOS 36.8 vs 21.3 months, *p* = 0.034 for liver metastasis, and 46 vs 30 vs 25 months for SBRT plus TKI, whole-brain radiotherapy [WBRT] plus TKI and TKI alone, respectively (*p* < 0.001)).

However, most of these studies were developed before the advent of osimertinib, which is more effective than first-generation anti-*EGFR* TKIs, possibly changing the magnitude of benefit of LAT used upfront.

In a single-arm, phase II trial (N = 43), patients received LCT with SABR after 8 weeks of osimertinib (if at least stable disease was achieved) [54]. Median PFS was 32.6 months and mOS 45.7 months. However, it is important to note that the inclusion criteria did not report a maximum number of metastatic lesions.

Regarding *ALK*-positive disease, the only data available about the use of LAT are for oligoprogressive disease and for diffuse metastatic NSCLC (see paragraph below).

Borghetti et al. investigated the role of conventional radiotherapy and SBRT in patients with *EGFR*-mutated or *ALK*-rearranged stage IV NSCLC [55]. A total of 50 patients were enrolled and received radiotherapy concomitantly, previously or after TKIs (only crizotinib for *ALK*-rearranged disease). The median OS was 19.3 months, and the group with SBRT (18% of cases) showed a better OS at univariate analysis (*p* = 0.043). In the BRIGHSTAR single-arm phase I study (N = 34), patients with *ALK*-rearranged NSCLC (not all oligometastatic) received brigatinib for 8 weeks followed by LCT (radiation or surgery) [56]. PFS at 3 years was 66%.

Notably, in oncogene-addicted disease, due to the probable persistence of micrometastatic disease even after LAT to all apparent sites, the continuation of TKI after LAT would be mandatory. This issue, combined with the advent of new generations of potent TKIs, weakens the upfront or consolidative radiotherapy as the standard approach. ESMO guidelines do not recommend local treatments with radical intent for *EGFR*-mutated and *ALK*-rearranged metastatic disease if it is not in the oligoprogressive scenario (see paragraph below), though the level of evidence is low due to the lack of randomised data.

Specific studies for other drivers (*BRAF*, *RET*, *MET*, *KRAS*, *NTRK*) are lacking. However, ESMO guidelines offer the same recommendations for all oncogene-driven scenarios in which TKIs are available [14]. A balance in terms of side effects should always be taken into account, especially when considering the treatment of CNS lesions in patients in which TKI with high ORR can be used. In terms of safety, the synergistic effect on potential pulmonary toxicity given by radiotherapy and TKI must be taken into account [57]. Clinical trials specifically addressing this topic are ongoing and might provide crucial data to aid clinicians in choosing the best course of treatment for every single patient (Table 3).

### 2.4. Oligoprogressive Disease in Patients with omNSCLC and AGAs

In oncogene-addicted omNSCLC, local treatment is of particular importance in oligoprogressive disease, because oligoprogression might be the result of the development of a resistance mechanism in a specific site while all the others remain sensitive to the drug [58]. Prolonging the PFS of TKIs is crucial, since for many AGAs there are not many targeted treatments available. Local treatment using SBRT, surgery or others can be used to eradicate TKI-resistant subclones enabling prolonged TKI treatment, which may lead to increased PFS and overall survival.

ESMO guidelines underline the importance of incorporating the indication for oligoprogressive disease in every one of their algorithms [14], suggesting eradicating the few sites of progression and continuing the systemic treatment whenever deemed appropriate after a multidisciplinary evaluation. However, the evidence supporting this statement is not strong due to lack of robust data and dedicated RCTs.

Prolonging systemic treatment through maintenance of clinical benefit has always been an area of interest since the introduction of the first TKIs. However, only small studies or retrospective analyses are available, often interrupted due to the advent of newer, more potent generations of TKIs and slow accrual [59,60,61]. In a phase II trial, 8/12 patients with *EGFR*-mutated oligoprogressive NSCLC during treatment with osimertinib received LAT, with a PFS of 2.3 months [62].

In a multicentre prospective observational study of 583 patients with EGFR-mutated NSCLC treated with first line osimertinib, oligoprogression was the pattern of progression in 45.4% of patients. Patients with oligoprogression had a better OS than those with CNS PD and multiorgan PD (21.4 months vs 9.0 vs 11.3, respectively). Approximately half of them continued on osimertinib (49.4%), but these patients seemed to have a worse prognosis than those with oligoprogression who changed systemic treatment (16.7 vs 32.5 months, HR 2.5 *p* = 0.0009). However, it is not specified if patients who continued osimertinib beyond oligoprogression received LAT to PD sites [63].

In patients with *ALK*-positive NSCLC, the use of LAT in extracranial oligoprogressive disease with continuation of crizotinib (N = 14/38) showed an improved PFS (17 vs 9.1 months), with local control rate of 86% 12 months after SBRT. Interestingly, in this single-centre experience, more than one course of LAT was considered if subsequent oligoprogression occurred [64]. The two-year OS was 72% in those who continued crizotinib for more than a year vs 12% in those who did not (*p* < 0.0001), hinting at the importance of continuing systemic treatment.

In a retrospective analysis (N = 52), *ALK*-rearranged patients with disease progression during crizotinib underwent salvage radiotherapy (N = 19), achieving a higher PFS2 than those who did not receive it (N = 33, 10.0 vs 6.0 months, *p* = 0.003) [65].

In a phase II trial, 27 patients with *EGFR*-mutated, *ALK*- or *ROS*-rearranged disease were candidates to SBRT for up to five metastatic lesions within 6 months of starting TKIs without signs of progression. mPFS was 14.7 months (8.3 to 46.4) and mOS 70.8 months (41.8 to NR) [66].

Specific RCTs using the most recent and effective TKIs are needed to fully comprehend the magnitude of benefit that can be derived from this strategy (Table 3). Upfront CNS radiotherapy might not be necessary, with new generations of TKIs that have been proven to be effective in this metastatic site irrespective of previous radiotherapy [67,68]. As previously mentioned, synergistic effects and possible toxicity must be taken into account when designing trials and evaluating our patients.

## 3. Current Challenges and Open Questions

The integration of LAT and systemic treatment is key to improving the outcomes of patients with omNSCLC. Many open questions remain, due to the lack of data from RCTs and the heterogeneity of retrospective data.

The first question is whether to use RT or surgery as LAT. There is no evidence about the superiority of one technique over the other in this setting, and technical feasibility and expected toxicity should guide the clinician’s choice after a multidisciplinary assessment [69,70]. Most studies explored the role of surgery and radiotherapy in patients with NSCLC and a single metastatic lesion, reporting a better survival in patients without the involvement of mediastinal lymph nodes [71,72] and with intra-thoracic metastasis vs extra-thoracic (5-year OS: 48.5% vs 23.6%) [71]. The involvement of mediastinal lymph nodes represent a particular challenge, for which cCRT has historically been considered, as in earlier stages [73].

SBRT was tested for the treatment of lung metastases, mostly in studies including metastases from different primary tumours. Rusthoven et al. [74] carried out a phase I/II trial evaluating SBRT in this setting, demonstrating local control rates of 96% at 2 years. Most patients in this study (71%) received systemic treatment as well. Similar outcomes were reported in another prospective observational multi-pathology study [75], with a higher survival rate in patients who responded to systemic therapy prior to SBRT (2-year OS rate 15% vs 55%, *p* = 0.0001). The increased risk of lung toxicity when ICB and SBRT are used in combination must be taken into account [76].

SBRT has the advantage of allowing clinicians to treat multiple sites, often simultaneously, and usually allows shorter systemic therapy interruptions compared to surgery. Another possible advantage of SBRT is the abscopal effect, which is thought to be correlated with the enhancement of CD8+ activity, cytokine release and general immunogenic signalling triggered by RT [11].

In general, SBRT is preferred over surgery when multiple organs are involved, although the maximum number of treatable lesions remains unknown.

The technique and dosage of radiotherapy are also crucial: SBRT appears to be the preferred option, and different studies, although retrospective, encourage a dosing of at least 60Gy to the primary site [77]. ASTRO guidelines recommend a biologically effective dose of at least 50Gy, preferably 75Gy when no systemic treatment is associated [12].

Factors precluding radiotherapy could be the location of the target, the volume of the field, previous irradiation, pre-existent interstitial lung disease or connective tissue disorders. In patients who will not receive SBRT, alternative ablative technique such as thermal ablation (i.e., cryotherapy, microwave, radiofrequency) may be an option [78], although SBRT should always be preferred when feasible.

**Figure 2:**
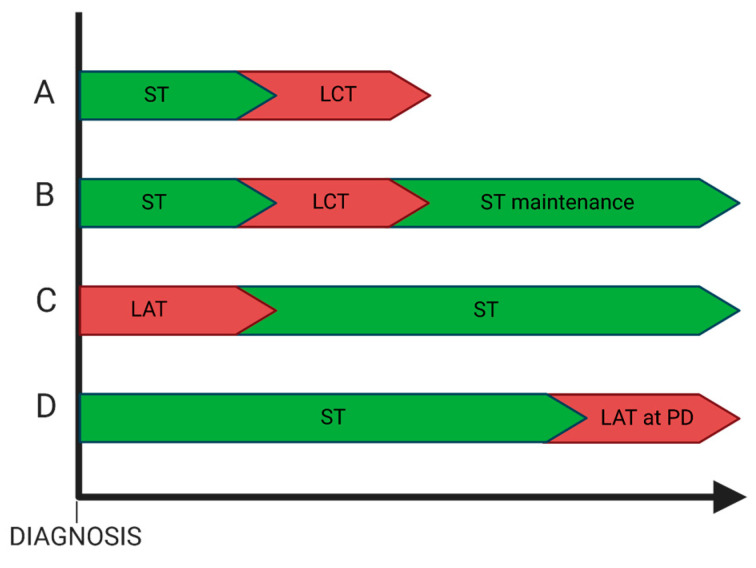
Representation of possible treatment sequencing in omNSCLC. A, B and C represent the possible treatment strategies for non-oncogene-addicted omNSCLC, where timing of local treatment depends on symptoms and metastatic sites and systemic treatment should involve immunotherapy. C and D represent the most appropriate options in omNSCLC with AGAs, where target therapies play a more prominent role. LAT and LCT could mean both surgery and radiotherapy. Abbreviations: ST = systemic treatment, LCT = local consolidative treatment, LAT = local ablative treatment, PD = progressive disease.

Another open question is the best treatment sequence and the best systemic treatment duration (Figure 2). The rationale for the use of systemic treatment upfront in omNSCLC is the immediate treatment of micrometastatic disease. Most of disease recurrences after local treatment occur in sites distant from those treated, and the risk remains high for several years, further suggesting the importance of systemic treatment [20,79].

Moreover, upfront systemic treatment will enable clinicians to select LAT for patients with a responsive disease. In this scenario, the particular case of oligoresidual disease in an initially multi-metastatic patient must be carefully evaluated, taking into account the different biology of the disease. Some of these patients were included in the aforementioned clinical trials of omNSCLC and LCT, but dedicated trials are crucial [12].

Both Bauml et al.’s [31] study and the SABR-COMET [22] trials explored the up-front use of radiotherapy, demonstrating its efficacy. However, in the survival curves, standard and experimental arm lines did not separate in the first months, suggesting that not all patients benefit from local treatments. As a consequence, most international guidelines recommend consolidative RT after having achieved at least a stable disease with systemic therapy, reserving up-front RT in case of symptomatic lesions or if the patients refuse and wish to delay systemic treatments.

Regarding the duration of systemic treatment after LAT, oncogene-addicted disease guidelines recommend continuing systemic treatment until multi-site progression, since LAT is predominantly only used to control resistant subpopulations [14].

For non-oncogene-addicted disease, ongoing clinical trials (NCT05255302 and NCT06219317) are investigating the optimal duration of maintenance treatment, and whether this would be necessary. Until further evidence is presented, two years of ICB and maintenance therapy with pemetrexed in adenocarcinomas remains the standard of care [13].

Liquid biopsy and circulating tumour DNA (ctDNA) could help to identify minimal residual disease after local treatments or to select higher risk patients who could benefit from continuing systemic treatment [80]. For instance, a recent multi-institutional cohort study of 1487 omNSCLC showed how undetectable ctDNA prior to radiotherapy was associated with a better PFS (mPFS of 5.4 vs 8.8 months (*p* = 0.004, HR = 1.57, CI 1.15–2.13) and OS (16.8 vs 25 months (*p* = 0.030, HR = 1.65, CI = 1.05–2.61) [81]. The dynamic ctDNA changes during treatment seem to correlate with response [82], so its monitoring could help assessing ICB and target therapy efficacy and select patients who could benefit from discontinuation or intensification of treatment [82,83,84]. Larger, prospective studies are needed to better understand the possible critical role of liquid biopsy and ctDNA in this setting.

## 4. Conclusions

Oligometastatic NSCLC is a specific entity within advanced NSCLC. A shared, internationally accepted definition, such as the one proposed by EORTC, should be a starting point for developing clinical trials and robust evidence to assess the best treatment for patients with omNSCLC. Multidisciplinary approaches are always recommended to ensure all treatment options are evaluated. In non-oncogene-addicted omNSCLC, systemic treatment should be primarily considered, reserving LCT to non-progressive patients. Conversely, timing of LAT in oncogene-addicted omNSCLC must be carefully evaluated during multidisciplinary discussions to determine whether to reserve it for oligoprogression or use it upfront. This also depends on the type of AGA, the site of the lesions and the symptoms of the patients.

As for LAT techniques, surgery and SBRT remain the most effective and safest options. Whenever available, these patients should be enrolled in clinical trials.

In the near future, ctDNA and other circulating biomarkers will hopefully become available with specific indications in omNSCLC to better select and monitor our patients.

## Figures and Tables

**Table 1:** Main ongoing clinical trials for patients with non-oncogene-addicted omNSCLC. Abbreviations: M = metastasis, CT = chemotherapy, RT = radiotherapy, SBRT = stereotactic body radiotherapy, ICB = immune checkpoint blockers, LCT = local consolidative treatment, SABR = stereotactic ablative radiotherapy, SoC = standard of care, adj = adjuvant, NS = not specified.

**STUDY**	**PHASE**	**Max *N* of M Sites/Organs**	**Investigational Treatment**	**Control Arm**
NCT05055583	2	3 m, 1 organ	Toripalimab + CT followed by surgery and adj toripalimab	Single arm
NCT03705403 (IMMUNOSABR2)	2	5 m	SABR or RT +/− ICB plus immunocytokine	SoC
NCT04767009	2	NS	ICB + SBRT	Single arm
NCT05472467 (IMCORT2)	2	5 m, 3 organs	Camrelizumab + CT + SBRT	Single arm
NCT03391869 (LONESTAR)	3	NS	ICB + LCT	ICB
NCT03774732 (NIRVANA LUNG)	3	NS	CT-ICB + RT	CT-ICB
NCT03721341 (SABR-COMET 10)	3	4–10	SoC + SABR	SoC
NCT03862911 SABR-COMET 3)	3	1–3	SoC + SABR	SoC
NCT03143322 STEREO-OS	3	3 (Bone only)	SoC + SBRT	SoC
NCT03955198	2	5 m, 3 organs	omNSCLC during durvalumab consolidation→SBRT	Single arm
NCT05846646	2	≤6	PULSAR-ICB + IMSA101	PULSAR-ICB
NCT05278052	3	5 m	LCT RT + SoC	SoC
NCT06141070 (ANDROMEDA)	3	5 m	SoC + SBRT	SoC
NCT05837052	2	3 m	serplulimab plus CT	Single arm
NCT04375904	2	1 lung	SABR for single pulmonary M	Sinlge arm
NCT05472467 (IMCORT-2)	2	5 m, 3 organs	Camrelizumab + SBRT and concurrent CT	Single arm
NCT03808337	2	5 m	SoC + SBRT	SoC
NCT05468242	2	NS	Neoadj CT-IO and bevacizumab followed by cCRT, followed by tislelizumab	Two cohorts, non-camparative
NCT05259319 (IMMUNOs-SBRT)	1	NS	Atezolizumab, Tiragolumab +SBRT	Singel arm
NCT02759783 (CORE)	2–3	3 m, 2 organs	SoC + SBRT	SoC
NCT06219317 (ICARS)	2	5 m, 3 organs	Cemiplimab +/− CT (4 cycles) plus LCT followed by cemiplimab maintenance	Cemiplimab +/− CT (4 cycles) plus LCT followed by placebo

**Table 2:** Summary of real-world data on immunotherapy for omNSCLC. Abbreviations: CRT = chemoradiotherapy, LAT = local ablative treatment, pCR = pathologic complete response, PFS = progression-free survival.

**Outcomes**	**Treatment Regimens**	**Number of Patients**	**Study**
5-year survival rate 28.3%, 4-year PFS 13%	Concomitant CRT	52	Guberina M. et al. [38]
pCR 54%	Chemoimmunotherapy followed by resection/LAT	13	Boch T. et al. [39]
PCR 46.2%, 2-year PFS 68.1%, 2-year OS 87.2	Chemoimmunotherapy followed by resection/LAT	26	Faehling M. et al. [40]
PFS 19 months, mOS 19.3 months	Immunotherapy plus LAT	68	Jongbloed M. et al. [41]
MOS 34.4 months	Immunotherapy plus LAT	218	Wiesweg M. et al. [42]
HR for OS vs chemotherapy 0.72 vs no immunotherapy (*p* < 0.001)	Immunotherapy	827	Ma J.C. et al. [43]

**Table 3:** Main ongoing phase II–III clinical trial for patients with oncogene-addicted omNSCLC. Abbreviations: M = metastasis, SBRT = stereotactic body radiotherapy, LCT = local consolidative treatment, NS = not specified, SoC = standard of care, TKI = tyrosine kinase inhibitor, LAT = Local ablative therapy, CNS = central nervous system, RT = radiotherapy, PD = progressive disease.

**STUDY**	**PHASE**	**Max *n* of M Sites**	**Investigational Treatment**	**Control Arm**
NCT04908956 (STEREO)	2	5 m	Osimertinib + SBRT to all sites	Single arm
NCT03410043 (NORTHSTAR)	2	NS	Osimertinib + LCT to at least 1 site	Osimertinib
NCT03827577 (OMEGA)	3	1–3	SoC (also non-oncogene-addicted) + LAT	SoC
NCT05277844 (TARGET-01)	2	5 m	EGFR or ALK TKI + LCT	EGFR or ALK TKI
NCT04643847	2	10 CNS	Upfront RT + Almonertinib	Single arm
NCT05167851 (ABLATE)	2	5 m	LAZERTINIB PLUS SBRT	Lazertinib
ISRCTN53398136 (HALT)	2–3	1–3 sites of PD, no CNS	TKI for actionable mutations + SBRT	TKI
NCT04405401 (SUPPRESS)	2	5 m,3 organs sites of PD	Systemic treatment plus SBRT	SoC
NCT 06014827	2	1–5 sites of PD, no CNS	RT/SBRT + osimertinib	Single arm
NCT03808662	2	1–5 sites of PD	SBRT	SoC
NCT06523673 (OPPRESS)	3	1–5 sites of PD, no CNS	SBRT + SoC	SoC
